# Sol-gel derived boehmite nanostructures is a versatile nanoplatform for biomedical applications

**DOI:** 10.1038/s41598-018-37589-1

**Published:** 2019-02-04

**Authors:** Yaroslav V. Solovev, Artur Y. Prilepskii, Elena F. Krivoshapkina, Anna F. Fakhardo, Ekaterina A. Bryushkova, Polina A. Kalikina, Elena I. Koshel, Vladimir V. Vinogradov

**Affiliations:** ITMO University, SCAMT laboratory 9, Lomonosova str., Saint Petersburg, 191002 Russian Federation

## Abstract

Alumina is one of the most promising carriers for drug delivery due to the long history of its usage as a vaccine adjuvant. Sol-gel synthesis provides excellent conditions for entrapment of biomolecules within an inorganic cage providing stabilization of proteins under the extremal conditions. In this paper, we show *in vitro* investigation of monodisperse alumina xerogel nanocontainers (AXNCs) using bovine serum albumin as a model protein entrapped in sol-gel alumina building blocks. Particularly, dose and cell-type dependent cytotoxicity in HeLa and A549 cancer cell lines were employed as well as investigation of antibacterial effect and stability of AXNCs in different biological media. It was shown, that the release of entrapped protein could be provided only in low pH buffer (as in cancer cell cytoplasm). This property could be applied for anticancer drug development. We also discovered boehmite nanoparticles effect on horizontal gene transfer and observed the appearance of antibiotic resistance by means of exchanging of the corresponding plasmid between two different *E. coli* strains. The present work may help to understand better the influence of AXNCs on various biological systems, such as prokaryotic and eukaryotic cells, and the activity of AXNCs in different biological media.

## Introduction

Targeted drug delivery systems gained much attention in past decades due to the necessity of enhancing treatment efficacy of socially significant diseases^1–10^. Sol-gel systems are one of the most promising platforms for drug delivery^11–13^. Even though most of the current works are devoted to silica-based sol-gel systems, it remains to be not approved by the FDA for intravenous, intramuscular and intraperitoneal administration^14–16^. In this regard, special attention to alumina, which is approved by the FDA for intramuscular injection, is deserved. Alumina is used as an adjuvant in antiviral and anticancer vaccines and can be easily obtained in sol-gel form by using alkoxide-based or inorganic salt routs^17–21^. Nevertheless, the activity of pristine sol-gel alumina building blocks, as well as alumina xerogel nanocontainers, have not been previously described in biological systems in details^22–24^. In this research, we call sol-gel alumina NPs as boehmite nanoparticles (NPs) in the reason of properties studying of nanoparticles that form stable alumina colloid. In our previous papers, we showed a promising approach to the creation of composite materials based on alumina and biomolecules by direct entrapment of the proteins within the sol-gel derived boehmite matrix (AlOOH)^25–27^. In this approach, the protein is immobilized in the xerogel boehmite cage during the room-temperature sol-gel transition^28,29^. It was shown, that it is possible to obtain systems both with complete immobilization of biomacromolecules and partial release of the proteins depending on the ratio of components and environmental conditions^26,27^. The entrapped proteins tightly interact with the walls of the ceramic matrix, which leads to an increase of their thermo- and photo-stability, so the structure of the immobilized protein can be preserved even at temperatures above the denaturation temperature under normal conditions^28–30^. Such a strategy of immobilization of protein molecules could be very promising in the development of hybrid systems with increased stability and shelf-life for the next generation of nanostructured drugs. However, detailed biological studies of the behavior of such systems *in vitro* and in different biological media have not been carried out so far.

In the current work, we study the effect of protein entrapment on the textural properties of AXNCs, the possibility of protein release in water, Ringer’s buffer, and low pH buffer (similar to the buffer of late endosomes in cells). The toxicities of boehmite NPs as well as of alumina xerogel nanocontainers were evaluated in HeLa and A549 cancer cell cultures. Cell survival, cell membrane damage, and the ability of the particles to be phagocyted by cells were also investigated. To make *in vitro* research more fulfilled we studied the effect of various concentrations of boehmite NPs on the growth of gram-negative *E. coli* and gram-positive *S. aureus*. In the literature, there are only fragmentary studies of antibacterial boehmite properties, some of which do not have statistical support^31,32^. In order to conduct a comprehensive analysis of boehmite NPs effects on microorganisms, we applied a set of methods, including the agar diffusion test, the colony-forming unit analysis (CFU/mL), and the compound effect on bacterial gene exchange. The latter study is of particular interest since it has shown that alumina leads to a significant increase of the plasmids conjugative junctions between different taxonomic groups of bacteria^33^.

## Results and Discussions

### Textural properties of boehmite NPs and AXNCs

**Figure 1:**
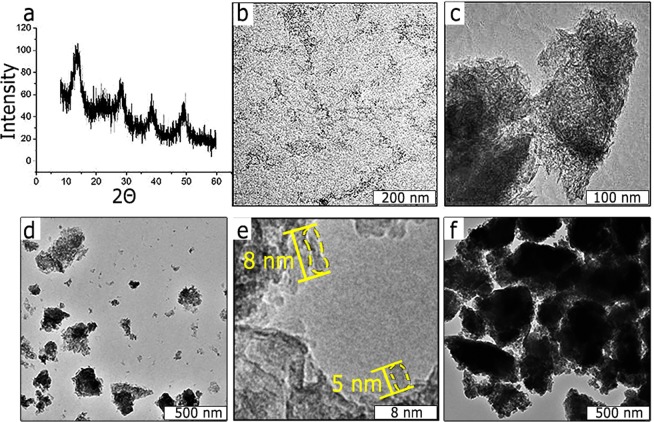
Textural properties of boehmite NPs and AXNCs: (**a**) XRD pattern (typical for boehmite structure); (**b**) TEM image of boehmite NPs in hydrosol; (**c**) TEM image of boehmite xerogel; (**d**) TEM image of AXNCs; (**e**,**f**) HRTEM and TEM images of BSA@AXNCs.

It should be noted, that the developed technique of obtaining of nanocrystalline AlOOH sol in aqueous solution under ultrasonic treatment implies the use of near-neutral pH values, which are comfortable for biomolecules and correspond to the range, where an optimal entrapment can be achieved. At these conditions, well-crystallized boehmite nanoparticles are formed (see Fig. 1a for X-ray diffraction (XRD) pattern, JCPDS file No. 21-1307) with the range of size between 5 and 8 nm (see high-resolution transmission electron microscopy (HRTEM) image, Fig. 1e), which was also confirmed by Scherer equation.

**Figure 2:**
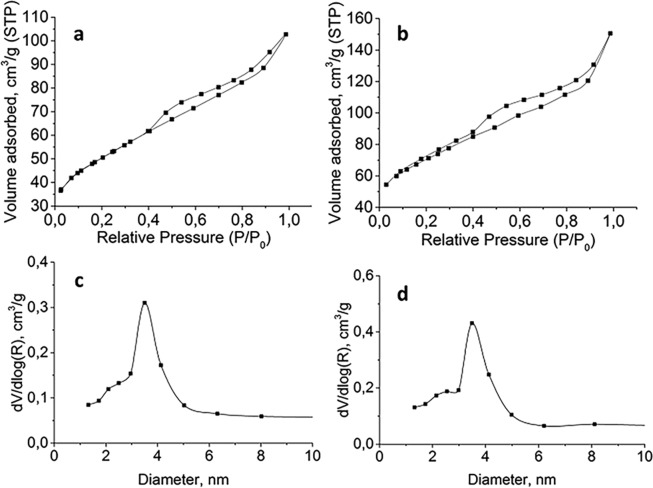
N_2_ adsorption-desorption isotherms and pore size distributions: (**a**,**c**) AXNCs; (**b**,**d)** BSA@AXNCs.

Among the six crystalline phases of alumina, only boehmite is biocompatible and also will be used for further biological tests. After physical sol-gel condensation of boehmite NPs (Fig. 1b), porous xerogel was formed (Fig. 1c). This matrix can be used for protein entrapment. After controlled grinding of xerogel, we can obtain quite monodispersed AXNCs (Fig. 1d). The similar results were obtained for BSA@AXNCs (Fig. 1e,f). HRTEM images of boehmite xerogel (Fig. 1c) and BSA@AXNCs (Fig. 1f) show almost identical textural properties, which were reflected in the data of nitrogen physisorption. We carried out nitrogen physisorption tests (Fig. 2) for free AXNCs (Fig. 2a,c) and with entrapped BSA (Fig. 2b,d) in order to study porous structure. According to IUPAC classification, the nitrogen sorption isotherms on samples belong to the Type IV isotherm given by most of the mesoporous adsorbents. Isotherms demonstrate hysteresis loops of Type H3 when the adsorption branch resembles a Type II isotherm, and the lower limit of the desorption branch is typically located at the cavitation-induced P/P_0_. As is known, loops of this type are given by non-rigid aggregates of rod-like particles (also confirmed by TEM image (Fig. 1c,f)). AXNCs containing BSA give effect on the type of sorption isotherm and the shape of the capillary-condensation hysteresis loop but textural parameters of individual and hybrid samples vary appreciably. For example, the specific surface area and pore volume for BSA@AXNCs exceed the values of free AXNCs by almost 1.5 times: 240 and 170 m^2^ g^−1^, respectively. Thus, we can talk about the disintegrating action of protein entrapped into alumina xerogel matrix. At the same time, the average pore size remains practically unchanged. The porous structure slightly changes from «bottle-like» to «cylinder-like», the percentage of 2.5 nm pores also increases.

Such a structural organization also influences the behavior of the entrapped proteins. As it was previously shown, proteins became much more stable and can operate at temperatures of complete denaturation of native proteins, even with enhancement of activity^25,29^. In this paper, it was not an exception. In our work, we measured the DSC spectra of pure lyophilized BSA and entrapped within AXNCs with 10% mass fraction. It was shown, that alumina renders the significant thermoprotective effect on BSA: the denaturation peak shifted from 48 °C to 84 °C. Our results correspond with previous data obtained on ovalbumin@alumina and ovalbumin@magnetite systems^29,30^. Moreover, we did not observe any significant decrease on thermoprotective properties with a mass fraction of entrapped BSA up to 50% (for the sake of brevity, the сurves are not represented here). These facts allow approving the safety of shelf-life of BSA@AXNCs that can be very important for the development of alumina-based drug delivery systems in the future. Especially, it would be winning property for various therapies, based on target protein delivery. This type of medicine approach is widely tested nowadays for treatment of many diseases such as Alzheimer, various types of tumors, bacterial and viral infections and others. Also, this approach can be successfully used in vaccinology. Safety of entrapped component (antigen) by room temperature in warm countries can decrease logistic expenses and losses of the vaccine samples in the reason of wrong storage conditions.

### Behavior of AXNCs in different biological fluids

**Figure 3:**
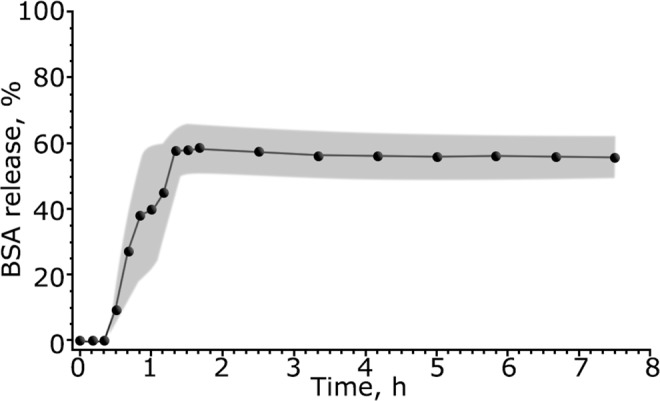
BSA release from AXNCs for 8 hours in low pH buffer (similar to late endosomal liquid) and Ringer solution. Points marked mean ± standard error (gray shading) of mean. The percentages indicated on the Y-axis are calculated from the equivalent amount of BSA in solution. No release of BSA was observed from AXNCs in distilled water and in Ringer’s buffer solution.

On the next step, we decided to investigate the behavior of AXNCs and BSA@AXNCs in different biological media. For this aim, we tested samples in low pH buffer (4.5) similar to late endosomal liquid, Ringer solution, DMEM culture medium, water and deionized water with RNA oligonucleotides. Surprisingly, we did not observe any release in solutions with near of neutral pH (water, Ringer solution) for BSA@AXNCs 10 wt.% of protein. At the same time, almost 60% protein release of entrapped BSA in low pH buffer was noted (Fig. 3). Taking into account negligible BSA release from AXNCs in deionized water and Ringer solution, we can suggest that BSA@AXNCs are chemically stable in extracellular liquid, but can be destroyed with drug release by protonation in late endosomes. Alumina matrix is quite inert for many chemicals, so the list of possible entrapped drugs could be very broad.

**Figure 4:**
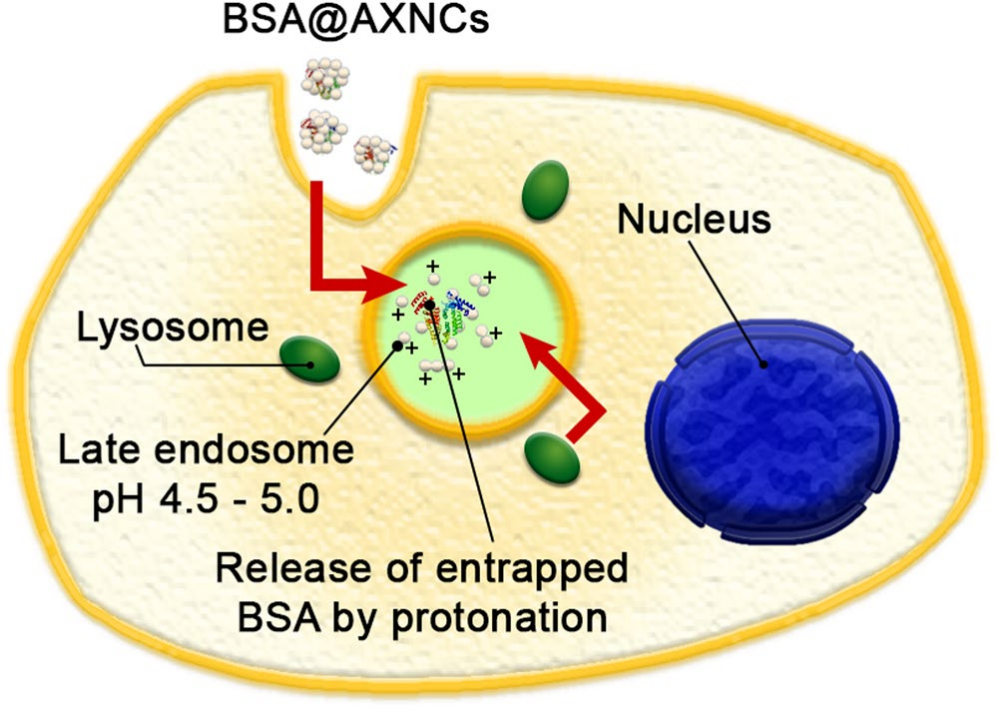
Principal theoretical scheme of penetration of BSA@AXNCs into cells and subsequent late endosome formation. Low pH environment contributes to the destruction of physically bonded nanoparticles by protonation and leads to release of the entrapped molecules (BSA) into the late endosome.

This observation makes drug@AXNCs promising for targeted delivery with phagocytosis-triggered drug release (Fig. 4). Moreover, AXNCs with entrapped chemical could be surely destroyed in the cytoplasm of cancer cells because their cytoplasm pH is significantly lower (approximately 5.5) in comparison with normal cells. It could be employed for additional selectivity for targeted anticancer drug delivery. Also, AXNCs can have significant pro-inflammatory (adjuvant) properties, similar to pristine boehmite currently used in vaccinology. Thus there is a big prospect for the use of drug@AXNCs system as antigen-delivery one.

**Figure 5:**
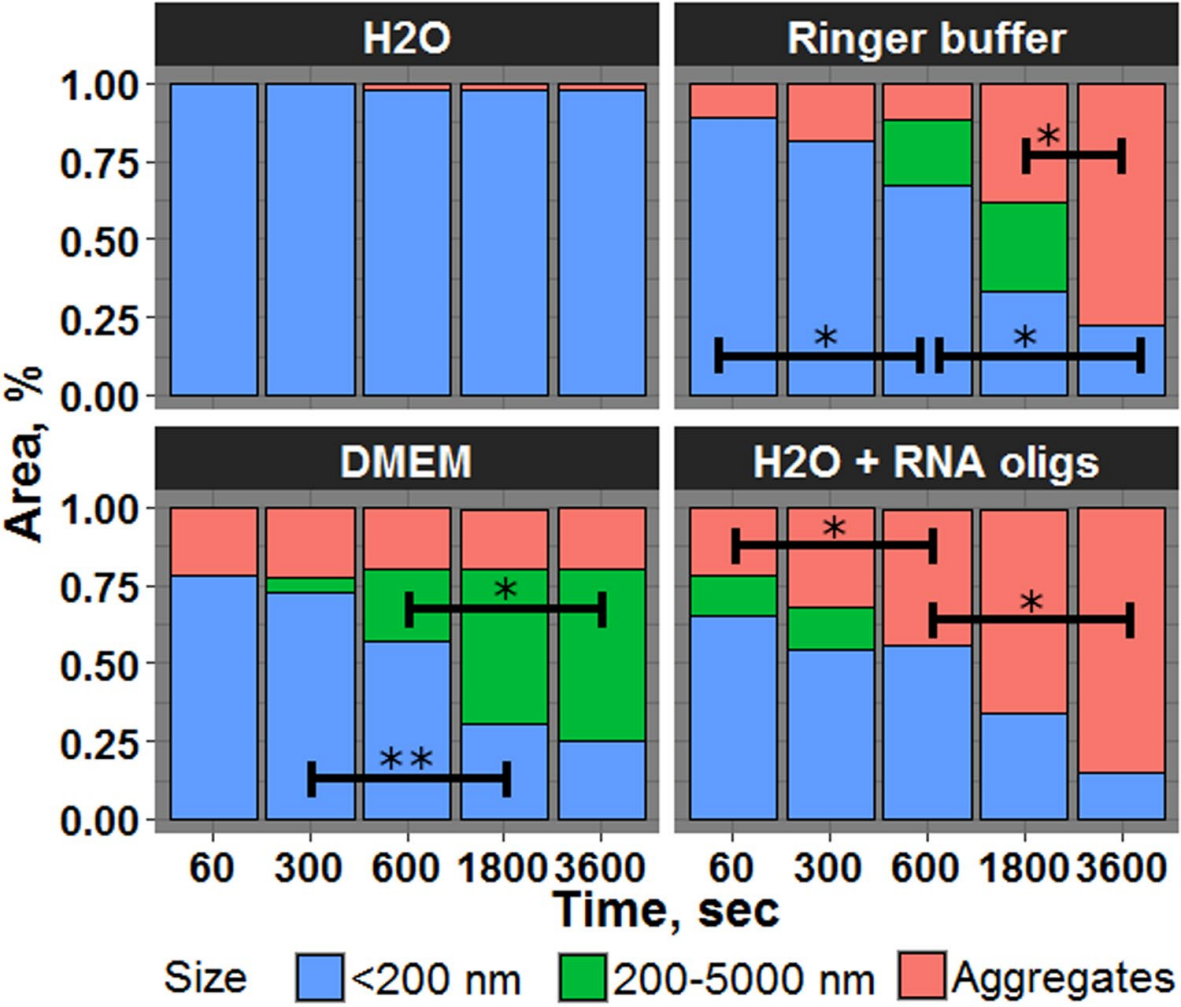
Sedimentation and aggregation stability of AXNCs in deionized water, Ringer buffer, DMEM and RNA oligonucleotides solution. *p < 0.05, **p < 0.01.

It was important not only to check BSA release from the alumina matrix but also to consider sedimentation and aggregation stability of AXNCs. The first experiments were conducted in deionized sterile water, and it was shown, that during the first 5 minutes system remains stable without notable aggregation (Fig. 5).

After that, negligible sedimentation was observed (approximately 1.5%) without further changes. It allows us to conclude that AXNCs do not aggregate in deionized water. Thus, we used the result of this experiment as a control. From the other side, the significant and rapid sedimentation in Ringer buffer was observed (Fig. 5). After 30 min more than 25% of all particles precipitated. Another 25% were aggregates of few AXNCs with size up to 5 microns. After 1 hour 75% of AXNCs precipitated. Ringer solution is very similar to human extracellular liquid. Thus, AXNCs form large aggregates after intramuscular injection. Theoretically, it can cause the list of problems, for example, the development of chronic inflammation due to inability to be phagocyted by antigen-presenting cells, mainly macrophages. In DMEM, the proportion of the precipitated AXNCs was constant during the whole experiment and percentage of aggregated AXNCs increased from 2.5% up to 50%, approximately, at the end of 1 hour. DMEM is widely used cell culture medium for eukaryotic cell cultures. Despite the fact, that the aggregates are unstable and can be destroyed by resuspension, this feature increases the cytotoxicity of AXNCs due to additional damage of cell membrane during various sample preparations (washing for MTT assay, detachment for flow cytometer, etc.). We also tested the possible interactions between AXNСs and RNA oligonucleotides. In previous articles, the possibility of interaction of alumina nanoparticles with phosphates and the positive influence of calcined alumina particles on the probability of horizontal gene transmission between different bacterial species were observed^23,33–35^. During experiments with RNA oligonucleotides we also observed sedimentation of AXNCs after addition of 0.5 mL of RNA oligonucleotides in deionized water in concentration 500 nM/mL. So, the percentage of precipitated AXNCs increased from 25% up to 80% to the end of 1 hour. This precipitate can be easily resuspended after 10 min treatment in an ultrasound bath. This fact can be used for increasing of bacteria transformation probability and should be taken into account. Antibacterial activity of boehmite NPs.

**Figure 6:**
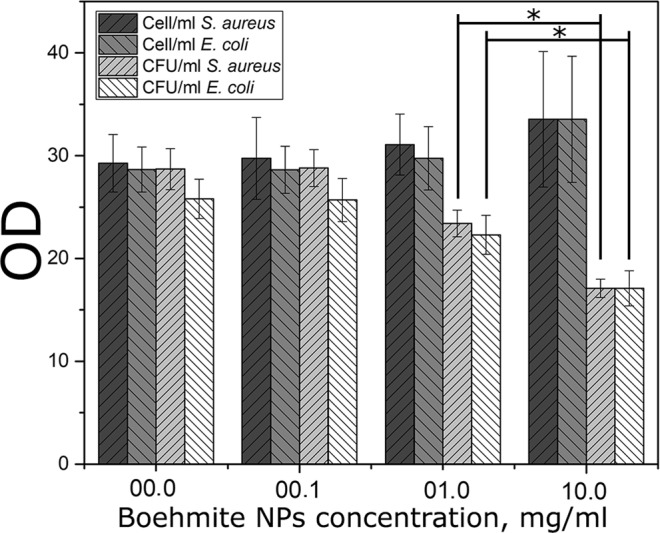
Antibacterial activity of boehmite NPs against *S. aureus* 209P и *E. coli* 292–116: cell per mL by optical density and CFU per mL by seeding on a dense nutrient medium. Data are presented as Mean ± StDev of three independent experiments: 0.1 mg/mL, 1 mg/mL and 10 mg/mL of boehmite NPs in bacterial suspensions of *S. aureus* and *E. coli*, respectively. Obtained data were compared with the сontrol experiment, *p < 0.05.

**Figure 7:**
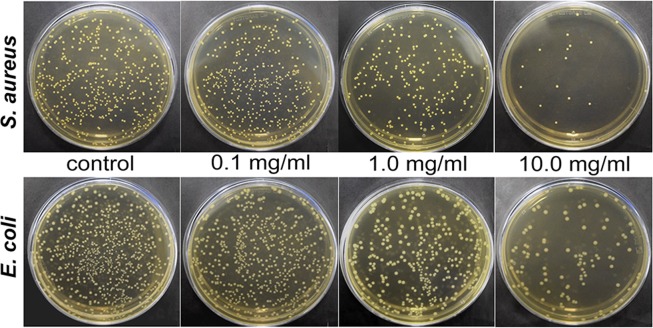
The results of CFU/mL analysis *S. aureus* 209P and *E. coli* 292–116 after incubation with boehmite NPs: seeding on a dense nutrient medium. Concentration of boehmite NPs: 0.0, 0.1, 1.0 and 10 mg/mL.

The effect of boehmite NPs on bacteria was evaluated on gram-positive (*S. aureus*) and gram-negative (*E. coli*) species. Boehmite NPs were tested at concentrations of 0.1, 1.0 and 10.0 mg/mL. Experiments of the density of culture change showed a small increase in density of the bacterial suspension, in particular, with the addition of boehmite NPs in the concentration of 10 mg/mL (Fig. 6). However, seeding this suspension on a dense nutrient medium showed an inverse relationship: a slight concentration decreases at boehmite concentration of 1 mg/mL and a sharp drop in cell viability at the concentration of 10 mg/mL (Figs 6 and 7).

**Figure 8:**
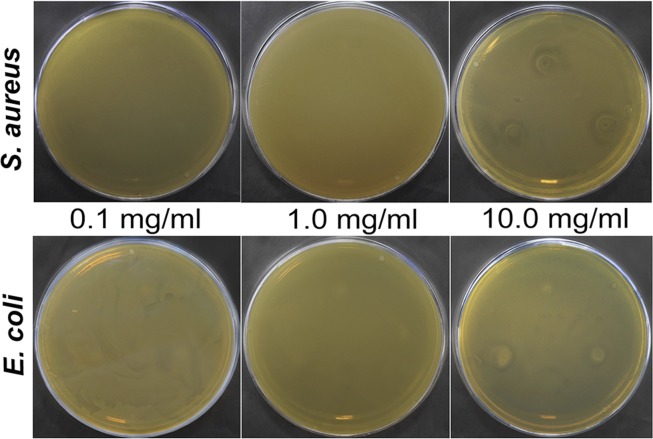
The results of the agar diffusion test of boehmite NPs: inhibition zones on bacterial lawns *S. aureus* 209P and *E. coli* 292–116. Concentration of boehmite NPs: 0.1, 1.0 and 10 mg/mL.

This correlates with the results of the agar diffusion test: there is an inhibitory zone (1–3 mm) when boehmite NPs are added in the concentration of 10 mg/mL (Fig. 8).

This can be explained by the fact, that boehmite NPs bind to fragments of destroyed cells in the process of normal growth. As a result, conglomerates, which have the same absorption as whole cells, are formed. This creates an additional dispersion, which leads to an increase in density. This important observation can explain the contradiction in the literature regarding the effect of nanostructures on cells.

The results of our study demonstrate that boehmite NPs have no significant antimicrobial effect on bacterial cells in a concentration of up to 10 mg/mL. In general, these data are consistent with the results described in the article by Amoura *et al*., where it was shown, that *E. coli* has almost normal growth in the presence of boehmite NPs in concentrations from 0.05 to 0.5 mg/mL, but it is strongly inhibited at concentration of 5 mg/mL^32^. Our data also showed a greater sensitivity of *S. aureus* to boehmite NPs than of *E. coli*, which is also in agreement with the fact, that usually, gram-positive bacteria are more sensitive to alumina nanoparticles than gram-negative bacteria^36–38^.

### Effect on plasmid horizontal junctions with boehmite NPs

For analysis of boehmite NPs effect on plasmid horizontal junctions, two-parent crosses were made between *E. coli* strains containing different plasmids with different resistance cassettes.

**Figure 9:**
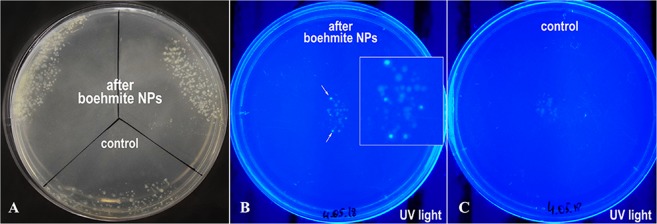
The results of plasmids’ horizontal junctions between *E. coli* 75-3 carrying plasmid P1 with a kanamycin and chloramphenicol resistance cassettes and *E. coli* XL1 Blue carrying plasmid pBADcycle3-mutant with ampicillin resistance cassette and GFP gene induced by arabinose promoter P_BAD_: transconjugant colonies, grown on a medium with three antibiotics (**A**) and with L-arabinose (**B,C**).

As a result of the study, the bacteria growth after incubation with boehmite was fixed on a medium with three antibiotics. This indicates that as a result of two parental mating, an isolate with resistance was obtained directly to ampicillin, kanamycin, and chloramphenicol. This may be due to the horizontal junctions of antibiotic resistance genes between the species of bacteria. Additional confirmation of the successful transfer of plasmids is green fluorescence of the colonies due to the synthesis of GFP (green fluorescent protein) from plasmid upon the addition of L-arabinose to the medium (Fig. 9B).

As can be seen in Fig. 9, the efficacy of conjugative plasmid transfer is slightly higher in the presence of boehmite NPs than in their absence.

Thus, the addition of boehmite NPs can enhance the effect of gene junction horizon between bacteria. This can be of great importance for understanding the mechanisms of the effects of NPs on cells. These are the first results indicating that boehmite can influence the horizontal transfer of genes. In the literature, there is only the one work, where the similar results were obtained on a commercial powder of alumina particles, the exact phase modification of which is unknown^39^. In this study, preliminary data were obtained of the effect of boehmite NPs on plasmid horizontal junction. Further, this problem certainly deserves a separate detailed study.

The effect of boehmite NPs on the promotion of horizontal transfer of antibiotic multiresistance genes between bacteria is essential for guiding the manufacture and application of nanomaterials in the environment and the evaluation of the possible consequences in ecology and biomedicine. The use of boehmite NPs in medicine should be carefully evaluated so as not to pose a threat to human health and the environment.

### Cellular tests

**Figure 10:**
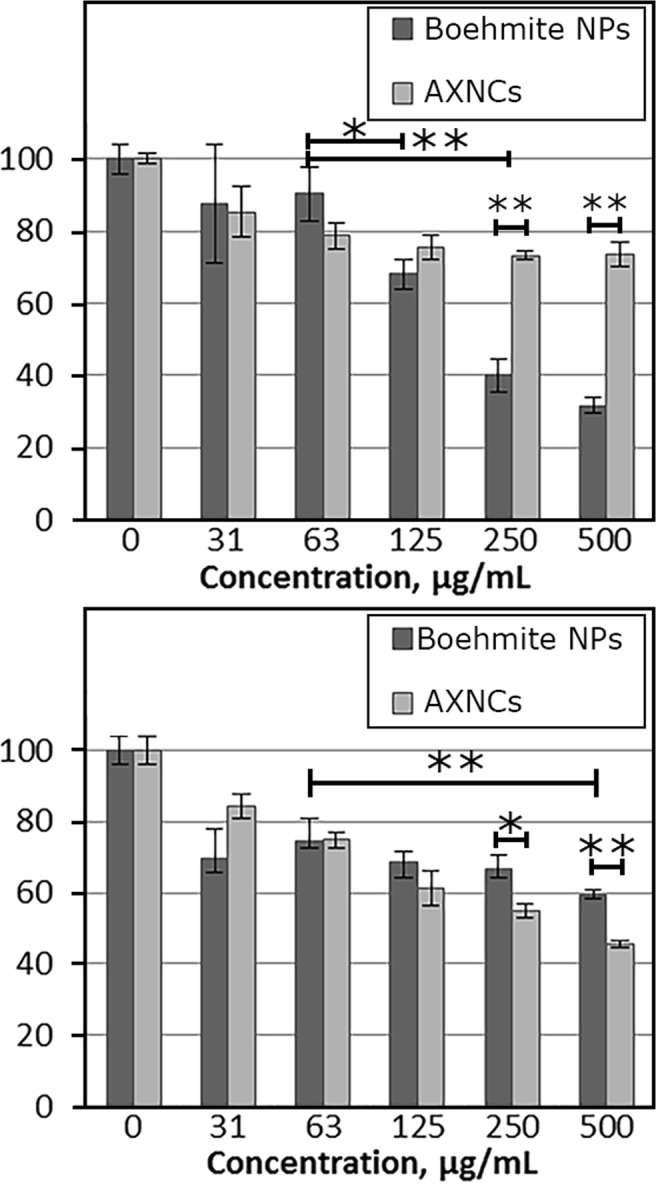
Cytotoxicity for (**a**) HeLa and (**b**) A549 cells after treatment with AXNCs and boehmite NPs for 72 h. Mean values of 3 measurements with standard deviation bars are shown (*p < 0.05, **p < 0.01).

To evaluate the biocompatibility of AXNCs and boehmite NPs, we treated HeLa and A549 cells with different concentrations of samples (31–500 μg/mL) for 72 hours and then made MTT assay. Figure 10 shows that in low concentrations (31 µg/mL and 63 µg/mL) the cytotoxicity is quite similar for HeLa and A549 cell lines, and there is negligible difference between AXNCs and boehmite NPs.

For HeLa cell line it was not observed any significant correlation between AXNCs concentration from 63 µg/mL to 500 µg/mL. Despite that, the significant decrease of survival of cells after addition of boehmite NPs in concentrations from 125 µg/mL to 500 µg/mL was detected. Boehmite NPs (Fig. 10a) were much more toxic for HeLa cells in comparison with AXNCs in high concentrations. In concentrations of 250 µg/ml and 500 µg/ml, the difference is statistically significant (p < 0.01). For A549 cell line we observed a correlation in cytotoxicity between AXNCs and alumina NPs in concentrations from 63 µg/mL to 500 µg/mL. According to the data obtained, boehmite NPs had an effect, which is approximately equal to AXNCs for A549 cells in concentrations from 63 µg/mL to 250 µg/mL. In concentration 500 µg/mL the difference is statistically significant (p < 0.01). Calculated IC_50_ (inhibitory concentration) for boehmite NPs and HeLa cells was about 190 µg/mL. For A549 cell line IC_50_ for AXNCs was about 400 µg/mL.

**Figure 11:**
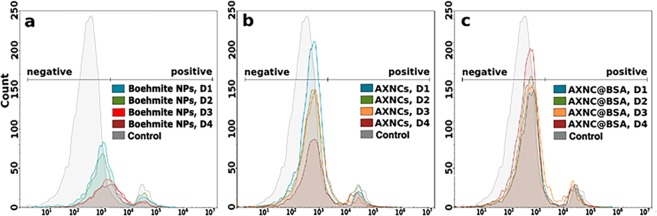
Comparison of fluorescence from 488 nm excitation of A549 cells stained by propidium iodide after 72-h incubation with the different concentrations (D1–D4) of boehmite NPs (**a**), pure AXNCs (**b**) and BSA@AXNCs (**c**). The relative fluorescence from PI is represented as a log scale. Gate PI-unstained cells [negative] or PI-stained cells [positive] used to analyze the cytotoxicity of nanoparticles. The difference between PI-negative and PI-positive cells was statistically significant with significance p < 0.05.

In general, we did not observe any strong pronounced cytotoxic effect and there is no concentration correlation between boehmite NPs and AXNCs. Various cancer cell lines showed the different response on NPs and AXNCs, and it could be determined by differences in cell metabolisms and gene expression in two observed cell lines. We reproduced obtained MTT results by flow cytometer on A549 cell line, which showed a higher sensitivity as a result of MTT. To estimate the cytotoxicity of nanoparticles, cells were incubated with the set of different concentrations of boehmite NPs (Fig. 11a), pure AXNCs (Fig. 11b) and BSA@AXNCs (Fig. 11c). These were added to A549 cell line: 16 μg/mL (D1), 32 μg/mL (D2), 65 μg/mL (D3), 130 μg/mL (D4) and were stained with propidium iodide (PI) as described in “Materials and Methods”. PI can penetrate only through damaged cell membranes, thus fluorescence intensity of propidium iodide directly correlates with the number of dead cells in the samples. For each sample, results were obtained as a one-parameter histogram with a count of cells on the Y-axis and the intensity of PI fluorescence on X-axis. To compare the results, histograms within each set of concentrations were overlaid. All cytotoxicity effects in subsequent experiments were described relatively the control data, which is shown as a gray curve in all graphs. The histograms overlay for all three sets (D1–D4) of nanoparticles does not show a significant difference of the PI-fluorescence. There is no histogram shift along the X-axis from the negative region corresponding to the control cells in the positive region, where the fluorescent cells should be located. However, a dose-dependent change in Y value for AXNCs and especially for pure boehmite NPs indicates the decrease in the absolute number of cells in the analyzed samples after 72 hours of incubation with nanoparticles.

According to the obtained results, boehmite NPs decreased the number of detected cells much more in comparison with AXNCs and BSA@AXNCs in all observed concentrations. Cytotoxic effect of boehmite NPs in concentrations D1 and D2 was similar, as well as in concentrations D3 and D4. However, the toxic influence of the D1 and D2 concentrations is significantly lower than D3 and D4 concentrations. For AXNCs and BSA@AXNCs we observed similar cytotoxicity in concentrations D2 and D3. In D4 concentration of BSA@AXNCs, the decrease of cytotoxicity was detected. It could be connected with the formation of large aggregates of BSA@AXNCs, that could not penetrate into the cells. This conclusion should be proved in future experiments. At the same time, the cytotoxicity of AXNCs in D4 concentration increased in comparison with lower concentrations as expected. The difference in D1 concentration between AXNCs and BSA@AXNCs is not statistically significant. The discrepancy between MTT results and flow cytometry data could be explained as a difference in measured properties of cytotoxic influence. MTT assay detected the decrease of activity of NAD(P)H-dependent cellular oxidoreductase enzymes in the electron transport chain in mitochondria and the flow cytometer detected the number of cells in the sample. Detection of propidium iodide dye helped us to determine the number of damaged cells but not the activity of NAD(P)H-dependent oxidoreductases. Thus, the results of two employed approaches complemented each other and allowed to understand better the mechanisms of cytotoxicity of synthesized nanoparticles.

**Figure 12:**
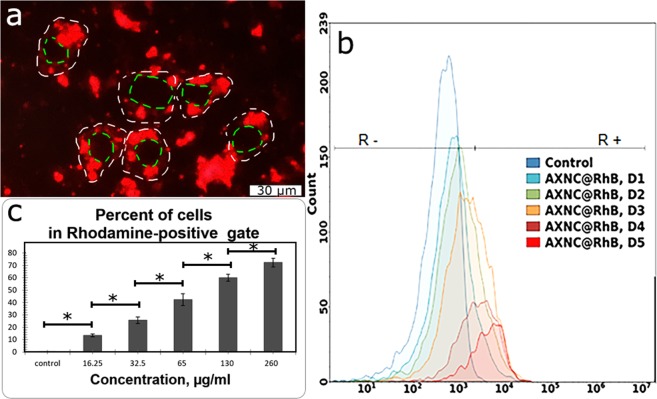
Dose-dependent penetration of RdB@AXNCs into A549 cells: (**a**) A549 cells stained with RdB@AXNCs, fluorescence microscopy (contour of cells marked with white dashed line, contour of nucleus marked with green dashed line), (**b**) cell fluorescence intensity after 72-h incubation with the different concentration of RdB@AXNCs (D1–D5), (**c**) percentage of cells in [R+] gate after incubation with different concentrations of nanoparticles. Gate RdB-unstained cells [R−] or RdB-stained cells [R+] used to analyze the penetration of nanoparticles into the cells. The relative fluorescence is represented as a log scale. The difference between D1 [R+] cells and D5 [R+] cells was statistically significant with significance p < 0.05.

On next step, it was essential to investigate penetration of potential drug carriers - AXNCs into cells. For this aim, A549 cells were incubated for 24 h in the presence of AXNCs conjugated with Rhodamine B (RdB@AXNCs). The efficiency of conjugation was evaluated by fluorescence microscopy (Fig. 12a). We observed accumulation of RdB@AXNCs inside the cell cytoplasm but not in the nucleus. Absense of NPs in cell nucleus is a typical result since nanoparticles mainly cannot penetrate the nucleus membrane. We also observed some compartmentalization of RdB@AXNCs which can indicate that they predominantly accumulated in endosomes.

**Figure 13:**
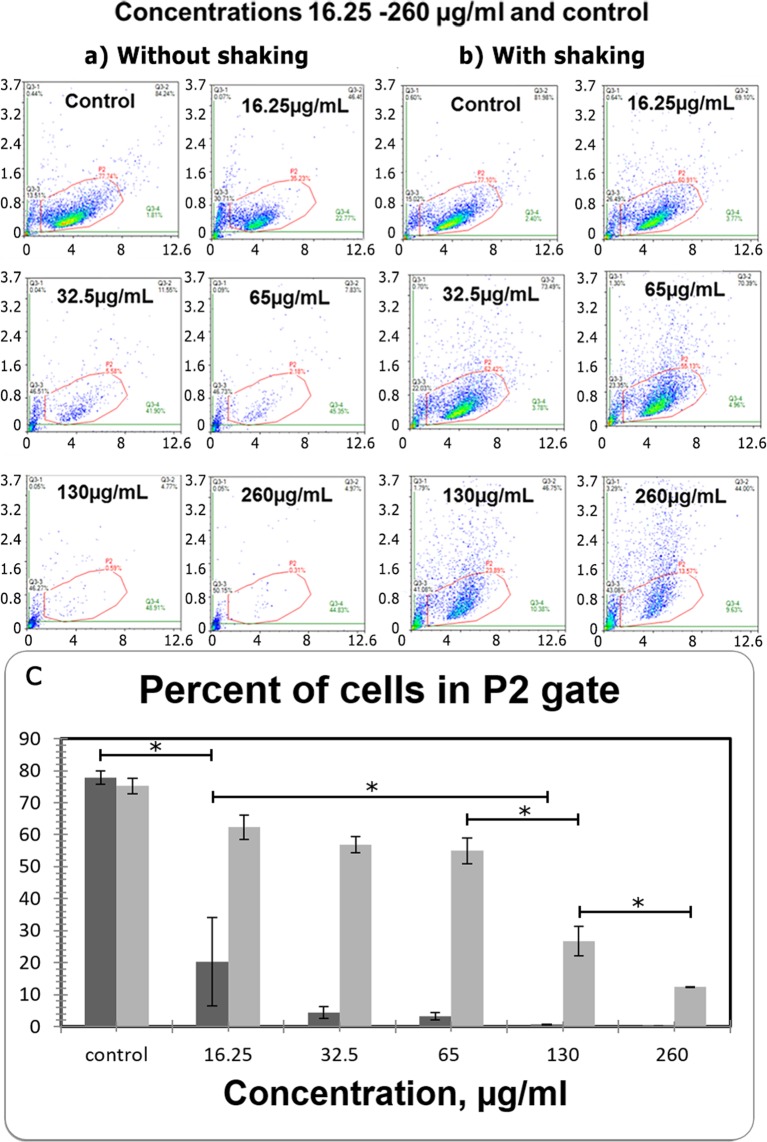
Cells sedimentation rate after adding different concentrations of AXNCs: without (**a**) or with (**b**) shaking. Cell count in “P2” gate plot: dark grey – with a single shake before measuring, light grey – with constant shaking at the measuring process (**c**). The “P2” gate means an area on the DotPlot, where the control cells are located. *p < 0.05.

From Fig. 12b it is clearly seen, that the dose-dependent shift of the histogram peak along the X-axis from [R−] gate to [R+] took place. It means the increase of the intensity of Rhodamine B fluorescence in the cells incubated with nanoparticles, which suggests about the permeability of RdB@AXNCs to cancer cells of the A549 line. Figure 12c shows, that after incubation with RdB@AXNCs in the concentration of 16.25 µg/mL only 13.5% of acquired cells had a fluorescent label, but already at a concentration of 130 µg/mL, the number of fluorescent cells reached 60%. This data demonstrates the high potential of AXNCs permeability for the observed A549 cell line, probably by phagocytosis. Indirectly dose-dependent particle penetration into A549 cell line is confirmed by the increased efficiency of cell settling after incubation with large doses of RdB@AXNCs (Fig. 13), that suggests about the increase of cell weight. In Fig. 13 a comparison of Dot Plot of the same samples measured under resting conditions (A) and constant shaking (B) is given. It can be seen from the Fig. 13c, that after the incubation with high concentrations of nanoparticles, the cells settle at the bottom of the plate and fall out of the measurement (a dependent decrease in the percentage of cells in “P2” gate). The fluorescence of cells after incubation with RdB@AXNCs at different concentrations (D1–D5) was measured twice in 24-well plate. We detected the significant increase of the cell weight after adding afore 16.2 µg/mL of RhB@AXNCs, and it was just impossible to detect cells without constant shaking of the plate by the flow cytometry. Thus, according to the obtained results, we detected two independent pieces of evidence, that the RhB@AXNCs penetrated into the A549 cells.

## Conclusions

In this article, the influence of boehmite NPs, AXNCs, and BSA@AXNCs on various biological systems was discussed. AXNCs, which consisted of boehmite building blocks, can be employed as the drug carriers with significant thermo- and chemoprotective properties. We discovered the possibility of the release of entrapped BSA from AXNCs in low pH solution similar to late endosomal. This trigger of the AXNCs destruction could be widely used in many cases, especially in vaccines and anticancer drug development. We also tested the behavior of boehmite NPs, AXNCs, and BSA@AXNCs in various liquids (deionized water, Ringer buffer and DMEM). We observed the formation of aggregates and sedimentation in Ringer solution, DMEM, and RNA oligonucleotides solution. It can cause different additional toxic effects, such as aggregation in extracellular space after intramuscular injection of AXNCs. At the same time, we did not detect any significant antimicrobial effect of boehmite NPs in concentrations up to 10 mg/mL, but the influence on horizontal gene transfer was found. We observed the appearance of antibiotic resistance by exchanging the corresponding plasmid between two different *Escherichia coli* stains after horizontal gene transfer. Thus, boehmite NPs could be used in biotechnology for enhancing the efficiency of gene transfection. The influence of gene transfection on eukaryotic cell lines and full organisms still needs to be researched.

In our article, we observed and described the interactions between alumina NPs and the prokaryotic and eukaryotic cells. A large number of additional *in vitro* experiments helped us to analyze the mechanisms underlying the observed results in *in vitro* tests. We discovered new properties of AXNCs that could be successfully employed in pharmacy, molecular biology, and material science.

## Experimental Section

### Chemicals

Aluminum isopropoxide (Sigma-Aldrich, CAS: 555-31-7), Bradford solution, 1–1400 µg/mL protein (Sigma-Aldrich, B6916-500 ml). MTT (3-(4,5-dimethylthiazol-2-yl)-2,5-diphenyltetrazolium bromide), propidium iodide (PI), bovine serum albumin (BSA) were obtained from Sigma-Aldrich. Dimethyl sulfoxide (DMSO) was from VWR, PBS tablets and fetal bovine serum (FBS) were purchased from Gibco.

### Cell cultures

A549 and HeLa cells (Biolot, Saint Petersburg, Russia) were maintained in DMEM (Dulbecco’s Modified Eagle Medium) and EMEM (Eagle’s Medium, Biolot) supplemented with 10% FBS and 50 µg/mL gentamycin (Biolot), respectively, at 37 °C in a humidified 5% CO_2_ atmosphere.

### Bacterial strains

For the antibacterial test, *Escherichia coli* 292–116 and *Staphylococcus aureus* 209P bacteria were used. For conjugation experiment with boehmite NPs treatment, *E. coli* 75–3 carrying plasmid P1 and *E. coli* XL1 Blue carrying plasmid pBADcycle3-mutant were used. P1 containing a kanamycin and chloramphenicol resistance cassettes, pBADcycle3-mutant containing ampicillin resistance cassette and GFP (green fluorescent protein) gene induced by arabinose promoter PBAD were employed.

### Synthesis of boehmite NPs

Aluminum isopropoxide was used for alumina sol synthesis^39^. For this aim, 3.3 g of aluminum isopropoxide powder was diluted in 50 mL of deionized water with following heating up to 85 °C and mixing at 500 rpm for 15 min. After that, the obtained suspended precipitate was treated by ultrasound (power = 260 W, frequency = 60 Hz) and simultaneously stirred over 160 min. The prepared alumina sol was stored in plastic vials at room temperature (approximately 22 °C).

### Preparation of alumina xerogel nanocontainers (AXNCs) and BSA@AXNCs

For the preparation of alumina xerogel nanocontainers, xerogel plates obtained by a physical condensation of boehmite sol were milled by a planetary ball mill (Fritsch, Germany). For this aim, agate mortar was used, diameter = 120 mm with 8 agate balls, diameter 10 mm. The system was treated for 20 min with frequency 800 rpm. The obtained powder was collected from agate mortar and agate balls by dint of metal spatula and tassel. AXNCs were further stored in sealed plastic test tubes at room temperature (approximately 22 °C). This process was used for the preparation of both empty nanocontainers and entrapped ones by BSA (BSA@AXNCs).

### Preparation of Rhodamine B labeled nanocontainers

We used Rhodamine B - labeled nanoparticles as a model object to determine the possibility of Rhodamine B to fluoresce while being entrapped in alumina xerogel nanocontainers (RhB@AXNCs). For the obtaining of RhB@AXNCs, we mixed 100 µl of 1% water solution of Rhodamine B with 10 mL of boehmite NPs sol. Finally, dehydration at room temperature and grinding to nanosized particles (~250 nm) took place.

### Measurement of sedimentation and co-aggregation of AXNCs in various biological liquids

In each experiment, 2 mg of monodisperse (d = 250 nm) AXNCs were employed. After dilution in 2 mL of liquids (deionized water, Ringer buffer, DMEM and in RNA oligonucleotides solution), the obtained suspension was studied by DLS analysis. The measurements were in 0, 300, 600, 1800 and 3600 seconds after the beginning of the experiment. The percentage of each group observed (<200 nm, 200–5000 nm, Aggregates) was used for distribution diagram’s creation. Group ‘Aggregates’ includes aggregated AXNCs of an unmeasurable size that forms a precipitate at the bottom of the cuvette.

### Protein release detection

Protein release from AXNCs in water, Ringer’s buffer and low pH buffer was studied. For this aim, 0.1 g of monodisperse AXNCs (d = 250 nm) was resuspended on Vortex in 1 mL of respective biological liquid. Right after resuspension, the suspension was placed in a cuvette for spectrophotometric measurements. After that, 1 mL of the commercial Bradford solution was added. The release was monitored in a kinetic mode during 8 hours by measuring light absorbance at wavelength 595 nm (standard for Bradford protein concentration measuring assay). All obtained data was collected and employed for subsequent statistical analysis.

### MTT assay

This test was used to evaluate the cytotoxicity of AXNCs and boehmite NPs. Cells at logarithmic phase of growth (5 × 10^3^/well) were plated into 96-well plates overnight and then treated for 72 h with samples dissolved directly in the culture medium (final concentrations 62.5–1000 µg/mL). After the completion of exposure, 200 µl of MTT solution in PBS (0.5 mg/mL) was added and the medium with NPs was discarded for 1.5 h at 37 °C at CO_2_ incubator. Then the MTT solution was aspirated, the formazan granules were dissolved in 200 µl DMSO.

### Flow cytometry

Flow cytometry was used to evaluate the necrosis in cells after the exposure of AXNCs, BSA@AXNCs, and boehmite NPs. Moreover, it was used to evaluate the AXNCs penetration into cells after the exposure of AXNCs conjugated with Rhodamine B (RdB@AXNCs). Cell morphology was estimated by FS/SS parameters. FS is called the forward-scattered light that is scattered in the forward direction, typically up to 20° offset from the laser beams axis. The FS intensity roughly equates to the cell’s and particle’s size. SS is called side-scattered light, measured approximately at a 90° angle to the excitation line. The SS intensity provides information about the granular content within a cell and a particle. Both FS and SS are unique for every cell and particle, and a combination of these was used to differentiate various cell types in a heterogeneous sample.

Fluorescence parameters of propidium iodide and Rhodamine B were measured separately in two different sets of experiments using 488 nm Excitation Laser and 615/20 nm emission filter. The data are shown as a histogram with the intensity of fluorescence along the X-axis and the number of fluorescent cells along the Y.

### Propidium iodide staining

A549 cells at logarithmic phase of growth (6 × 10^4^/well) were split into 24-well plates overnight and then treated for 72 h with boehmite NPs, pure AXNCs or BSA@AXNCs in four doses (62.5 µg/mL, 125 µg/mL, 250 µg/mL and 500 µg/mL respectively), dissolved directly in the culture medium and filtered through 0.42 µm filter. After the completion of exposure, cells were washed 2 times in PBS to remove the nanoparticles and detached from plates using Versene solution. Finally, cells were resuspended in 200 µL of PBS in concentration 3 × 10^5^/mL. Then 1 µL of propidium iodide (2 mg/mL) dye, which permeates only dead cells, was added to the cell suspension and this was incubated for 5 minutes. Samples were measured using the Novocyte Flow cytometer 3000 (ACEA Bioscience Inc., CA) and analyzed by the NovoExpress® software.

### Penetration of AXNCs into cells

The analysis of A549 cells at logarithmic phase of growth (6 × 10^4^/well) by flow cytometry was employed. These cells were split into 24-well plates overnight and then treated for 72 h with five doses of RdB@AXNCs (16.25 µg/mL, 32.5 µg/mL, 65 µg/mL, 130 µg/mL and 260 µg/mL respectively), dissolved directly in the culture medium and filtered through 0.45 µm filter (final concentration was determined by a previously made fluorescence calibration curve). After the completion of exposure, cells were washed for 2 times in PBS to remove the RdB@AXNCs, detached from plates using Versene solution and resuspended in 200 µL of PBS with a concentration of 3 × 10^5^/mL.

To obtain fluorescent images of A549 cells, 7 mg of the RdB@AXNCs powder was dissolved in 2 mL of PBS. Next, this solution was filtered through a 0.45 µm MCE filter (Biofil). A549 cells were grown for 24 hours up to 80% confluence. 200 µl of the filtered RdB@AXNCs solution was added to 2 mL of DMEM culture medium in 12-well culture plate (Eppendorf). Cells were incubated with RdB@AXNCs for 24 h. Before microscopic observation culture medium was discarded, cells were washed thrice with PBS. Fluorescent images were obtained at 400× magnification, green filter excitation.

### Antibacterial study of boehmite NPs

The effect of different concentrations of boehmite NPs was evaluated on bacteria *S. aureus* 209 P and *E. coli* 292–116. The nutrient medium was added to the flask with the addition of boehmite NPs at concentrations of 0.1, 1.0 and 10.0 mg/mL and the inoculum. The growth of bacteria was assessed by change in optical density of the cell suspension with the use of DEN-1B densitometer (BioSan, Latvia). Additionally, the resulting suspensions were seeded on a dense nutrient medium to determine the CFU/mL. Also, we used the agar diffusion test: 5 μl of boehmite NPs at concentrations of 0.1, 1.0 and 10.0 mg/mL were added to the grown lawn and incubated for 24 hours at 37 °C. At the end of the incubation, the inhibition zone of bacterial growth was fixed. The experiments were carried out in triplicate.

### Horizontal gene transfer with boehmite NPs

The effect of boehmite NPs on plasmid horizontal junctions between bacteria was studied on *E. coli* strains carrying plasmids with different resistance cassettes. The solution of boehmite NPs (5 mM) in the nutrient medium was added to the donor and recipient mixtures and stirred by shaking for 2 hours. After overnight incubation, the inoculum, which contained ampicillin, kanamycin, and chloramphenicol (200 μg/mL, 100 μg/mL, 12.5 μg/mL, respectively), was applied to a nutrient medium. Additionally, the effectiveness of plasmid horizontal junctions was tested by L-arabinose addition in the medium. As a result, transconjugants carrying both plasmids can be detected not only by resistance to three antibiotics but also by characteristic green fluorescence over UV- irradiation. In parallel, in each experiment, parental strains were inoculated on media with appropriate antibiotics, to which these cannot resist. The absence of growth confirmed the absence of the appearance of spontaneous antibiotic-resistant mutants.

### Characterization techniques

The adsorption and textural properties of samples were estimated from isotherms of low temperature (77 K) nitrogen physical adsorption-desorption via volumetry on Quantachrome Nova 1200e surface area and porosity analyzer. The specific surface area was determined by the BET method. The cumulative adsorption volume was in the range of pores from 1.7 to 300 nm, the average pore diameter and differential mesopore size distribution were calculated by Barrett-Joyner-Halendy method (BJH). Before the analysis, samples were held in a vacuum for 4 h. The relative error in determining the pore volume was ±1%, for the surface area and the pore size ±10%. The crystal phase and crystallinity of the samples were studied by X-ray diffraction method (Bruker D8 Advance) using Cu-Kα irradiation (λ = 1,54 Å), samples being scanned along 2θ in the range of 4–60° at a speed of 0.5 degrees per minute. The samples for transmission electron microscopy (TEM) were obtained by dispersing of a small probe in ethanol for forming a homogeneous suspension. Then, a drop of suspension was coated on a copper mesh covered by carbon for TEM analysis (FEI TECNAI G2 F20, at an operating voltage of 200 kV). Synchronous thermal analysis curves were obtained by 204 F1 Phoenix NETZSCH apparatus, and a heating rate of 10 °C min^−1^ was used from 30 °C to 150 °C in nitrogen. All prepared suspensions were also measured by dynamic light scattering (DLS) by Photocor EPM/Photocor Compact Z. For MTT assay, optical density was measured at 570 nm by Tecan Infinite 50 spectrophotometer (Austria). Cell viability was calculated as the percentage of optical densities in wells with each concentration of AXNCs and alumina NPs. The data were normalized to the optical density of untreated cells (100%). For flow cytometry, samples were measured using the Novocyte Flow cytometer 3000 (ACEA Bioscience Inc., CA) in 96-well plates (total volume 200 µL) with 1 cycle of mixing before each well acquired. The growth of bacteria was assessed by the change in the optical density of the cell suspension with the use of DEN-1B densitometer. The flask with the suspension was staged in a shaker-incubator at 37 °C under 200 rpm, and it grew for 12 hours.

## References

[1] Rosenblum D, Joshi N, Tao W, Karp JM, Peer D (2018). Progress and challenges towards targeted delivery of cancer therapeutics. Nat. Commun..

[2] Tao W (2013). Docetaxel-loaded nanoparticles based on star-shaped mannitol-core PLGA-TPGS diblock copolymer for breast cancer therapy. Acta Biomater..

[3] Tao W (2016). Polydopamine-based surface modification of novel nanoparticle-aptamer bioconjugates for *in vivo* breast cancer targeting and enhanced therapeutic effects. Theranostics.

[4] Tao W (2015). Blended nanoparticle system based on miscible structurally similar polymers: a safe, simple, targeted, and surprisingly high efficiency vehicle for cancer therapy. Adv. Healthcare Mater..

[5] Tao W (2014). Synthesis of cholic acid-core poly (ε-caprolactone-ran-lactide)-b-poly (ethylene glycol) 1000 random copolymer as a chemotherapeutic nanocarrier for liver cancer treatment. Biomater. Sci..

[6] Ding L (2017). Intracellular fate of nanoparticles with polydopamine surface engineering and a novel strategy for exocytosis-inhibiting, lysosome impairment-based cancer therapy. Nano Lett..

[7] Zhu X (2018). Intracellular mechanistic understanding of 2D MoS2 nanosheets for anti-exocytosis-enhanced synergistic cancer therapy. ACS Nano.

[8] Tao W (2017). Antimonene Quantum Dots: Synthesis and Application as Near‐Infrared Photothermal Agents for Effective Cancer Therapy. Angew. Chem..

[9] Tao W (2017). Black phosphorus nanosheets as a robust delivery platform for cancer theranostics. Adv. Mater..

[10] Tao, W. *et al*. Two‐Dimensional Antimonene‐Based Photonic Nanomedicine for Cancer Theranostics. *Adv. Mater*., 1802061, 10.1002/adma.201802061 (2018). 10.1002/adma.201802061 PMC7028391 30043416

[11] Böttcher H, Slowik P, Süß W (1998). Sol-gel carrier systems for controlled drug delivery. J. Sol-Gel Sci. Technol..

[12] Yong Q, Park K (2001). Environment-sensitive hydrogels for drug delivery. Adv. Drug Delivery Rev..

[13] Byeongmoon J, Kim SW, Bae YH (2012). Thermosensitive sol–gel reversible hydrogels. Adv. Drug Delivery Rev..

[14] Trewyn BG, Slowing II, Giri S, Chen H-T, Lin VS-Y (2007). Synthesis and functionalization of a mesoporous silica nanoparticle based on the sol–gel process and applications in controlled release. Acc. Chem. Res..

[15] Arcos D, Vallet-Regí M (2010). Sol–gel silica-based biomaterials and bone tissue regeneration. Acta Biomater..

[16] Lu J, Liong M, Zink JI, Tamanoi F (2007). Mesoporous silica nanoparticles as a delivery system for hydrophobic anticancer drugs. Small.

[17] Shen C (2017). Polyethylenimine-based micro/nanoparticles as vaccine adjuvants. Int. J. Nanomed..

[18] Katz JB, Hanson SK, Patterson PA, Stoll IR (1989). *In vitro* assessment of viral antigen content in inactivated aluminum hydroxide adjuvanted vaccines. J. Virol. Methods.

[19] Allison AC, Byars NE (1991). Immunological adjuvants: desirable properties and side-effects. Mol. Immunol..

[20] Noad R, Roy P (2003). Virus-like particles as immunogens. Trends Microbiol..

[21] Shapovalova, O. & Vinogradov, V. Ultrasound-Assisted Sol–Gel Synthesis of Nano-Boehmite for Biomedical Purposes, presented at ICNN 2015, Czech Republic, Prague, 9–10 July 2015.

[22] Hench LL (1997). Sol-gel materials for bioceramic applications. Curr. Opin. Solid State Mater. Sci..

[23] Pereira MM, Clark AE, Hench LL (1994). Calcium phosphate formation on sol‐gel‐derived bioactive glasses *in vitro*. J. Biomed. Mater. Res. Part A.

[24] Saravanapavan P, Hench LL (2001). Low‐temperature synthesis, structure, and bioactivity of gel‐derived glasses in the binary CaO‐SiO_2_ system. J. Biomed. Mater. Res. Part A.

[25] Vinogradov V, Avnir D (2014). Exceptional thermal stability of therapeutical enzymes entrapped in alumina sol–gel matrices. J. Mater. Chem. B.

[26] Vinogradov VV (2018). Composites Based on Heparin and MIL-101 (Fe): The Drug Releasing Depot for Anticoagulant Therapy and Advanced Medical Nanofabrication. J. Mater. Chem. B.

[27] Chapurina YE (2016). Streptokinase@alumina nanoparticles as a promising thrombolytic colloid with prolonged action. J. Mater. Chem. B.

[28] Shapovalova OE, Levy D, Avnir D, Vinogradov VV (2016). Protection of enzymes from photodegradation by entrapment within alumina. Colloids Surf. B.

[29] Rutenberg A, Vinogradov VV, Avnir D (2013). Synthesis and enhanced thermal stability of albumins@alumina: towards injectable sol–gel materials. Chem. Commun..

[30] Drozdov AS, Shapovalova OE, Ivanovski V, Avnir D, Vinogradov VV (2016). Entrapment of enzymes within sol–gel-derived magnetite. Chem. Mater..

[31] Rashed HH, Moatasemballah J (2017). Antibacterial Activity of Boehmite Nanoparticles Synthesized by Arc Discharge in Deionized Water Technique. Journal of Al-Nahrain University.

[32] Amoura M, Nassif N, Roux C, Livage J, Coradin T (2007). Sol–gel encapsulation of cells is not limited to silica: Long-term viability of bacteria in alumina matrices. Chem. Commun..

[33] Pan X (2010). Mutagenicity evaluation of metal oxide nanoparticles by the bacterial reverse mutation assay. Chemosphere.

[34] Qiu Z (2012). Nanoalumina promotes the horizontal transfer of multiresistance genes mediated by plasmids across genera. Proc. Natl. Acad. Sci. USA.

[35] Balasubramanyama A (2010). *In vitro* mutagenicity assessment of aluminium oxide nanomaterials using the Salmonella/microsome assay. Toxicol. In Vitro.

[36] Geoprincy G, Gandhi NN, Renganathan S (2012). Novel antibacterial effects of alumina nanoparticles on Bacillus cereus and Bacillus subtilis in comparison with antibiotics. Int. J. Pharm. Pharm. Sci..

[37] Sadiq IM, Chowdhury B, Chandrasekaran N, Mukherjee A (2009). Antimicrobial sensitivity of *Escherichia coli* to alumina nanoparticles. Nanomedicine.

[38] Mukherjee, A., Mohammed Sadiq, I., Prathna, T. C. & Chandrasekaran, N. Antimicrobial activity of aluminium oxide nanoparticles for potential clinical applications. Science against microbial pathogens: communicating current research and technological advances (Formatex Research Center, 2011).

[39] Shapovalova, O. E., Drozdov, A. S., Brushkova, E. A., Morozov, M. I. & Vinogradov, V. V. Room-temperature fabrication of magnetite-boehmite sol-gel composites for heavy metal ions removal. *Arabian J. Chem*., 10.1016/j.arabjc.2018.02.011.

